# Effect of In Situ Mg-Sialon on the Oxidation Behavior of Low-Carbon MgO-C Refractories

**DOI:** 10.3390/ma16051892

**Published:** 2023-02-24

**Authors:** Bo Dong, Chao Yu, Guangchao Xing, Jinghui Di, Jun Ding, Qingyou Zhu, Hongxi Zhu, Chengji Deng

**Affiliations:** The State Key Laboratory of Refractories and Metallurgy, Wuhan University of Science and Technology, Wuhan 430081, China

**Keywords:** MgO-C refractories, low-carbon, oxidation, Mg-sialon

## Abstract

The in situ Mg-sialon in low-carbon MgO-C refractories was studied with respect to its oxidation behavior and mechanism at 1500 °C. The results indicated that the oxidation index and rate constant of low-carbon MgO-C refractories with Mg-sialon were 26.2% and 0.51 × 10^−3^ cm^2^/min at 1500 °C for 2 h, respectively. The formation of a dense MgO-Mg_2_SiO_4_-MgAl_2_O_4_ protective layer contributed to considerable oxidation resistance, and the generation of this thicker layer was due to the combined volume effect of Mg_2_SiO_4_ and MgAl_2_O_4_. The reduced porosity and more complex pore structure were also found in the refractories with Mg-sialon. Therefore, further oxidation was restricted as the oxygen diffusion path was effectively blocked. This work proves the potential application of Mg-sialon in improving the oxidation resistance of low-carbon MgO-C refractories.

## 1. Introduction

Magnesia-carbon refractories have been widely employed as the linings of BOF/LD converters, electric arc furnaces (EAF), and refining furnaces because of their excellent resistance to thermal shock and slag corrosion [1,2]. The application of low-carbon refractories became one of the prime tasks due to the demand for advanced steel-making technologies [3,4], energy saving, and carbon neutralization in recent years [5,6]. However, the decrease in graphite content would increase thermal stress and impair corrosion resistance; meanwhile, a loose decarbonization layer was easily caused by the oxidation of graphite, further damaging the overall performance of MgO-C refractories.

Extensive studies have been explored to prevent the oxidation of graphite in unburned MgO-C refractories, such as the application of additives [7,8,9], the usage of anti-oxidation coating [10,11], and the modification of carbon source [12,13]. Meanwhile, the in situ formed oxide ceramic phases, like Mg_2_SiO_4_ or MgAl_2_O_4_, could influence the mechanical strength, thermal shock resistance, and oxidation resistance of MgO-C refractories [14,15,16]. In order to further enhance the service performance of low-carbon MgO-C refractories, the high-temperature nitridation of MgO-C refractories was performed, i.e., the in situ Si_3_N_4_ reinforced MgO-C refractories have been proven to be effective with their optimized mechanical properties and slag resistance [17,18,19]. In contrast, the oxidation resistance of MgO-C refractories with these nitride reinforcements has its limits [18,19].

Sialon belongs to the solid solutions of Al and O atoms in the crystal lattice of Si_3_N_4_ [20,21], and it has gained much attention due to its high flexural strength (350~1100 MPa) [22], low thermal expansion (3.7~4.5 × 10^−6^/°C) [23], and excellent resistance to oxidation and corrosion [24,25,26]. Two types of polymorphs of Sialon, α- and β-phases, were widely researched. In comparison, α-Sialon has higher hardness and better thermal shock resistance than β-Sialon, and its crystal has large interstices, which could accommodate some metal cations [25,26]. Previous research has shown that the physical and chemical properties of the material are regulated due to the point defects and lattice distortion caused by ion substitution [27,28,29]. With the doping of metal ions and the replacement of Al^3+^, the Eu-doped α-sialon, Fe-doped α-sialon, and Mg-doped α-sialon showed other unique properties, such as better optical properties, mechanical properties, or corrosion resistance [30,31,32]. In particular, Mg-doped α-sialon (also called Mg-sialon) has superior high-temperature strength due to a reduction in the intergranular glass phase and the achievement of the desired microstructure [33,34]. However, the oxidation behavior of low-carbon MgO-C refractories within in situ Mg-sialon has never been performed and investigated.

In this work, the oxidation behavior of the Mg-sialon reinforced low-carbon MgO-C refractories was discussed by analyzing phase and microstructure evolution, and the oxidation mechanism of Mg-sialon in MgO-C refractories was also studied.

## 2. Materials and Methods

### 2.1. Raw Material

Fused magnesia particles (1–3 mm, 0.074–1 mm, and <0.074 mm, ~98 wt% MgO; Gongyi, China), flake graphite (≤0.074 mm, ~99 wt% C; Gongyi, China), silicon powder (≤0.074 mm, ~99 wt% Si; Xingtai, China), and Al_4_SiC_4_ powder (<0.074 mm, ~98 wt% [35]) were used as the raw materials. The composition of the samples is listed in Table 1. The batch containing 67 wt% magnesia aggregate, 26 wt% magnesia powder, 4 wt% flaky graphite, and 3 wt% Si powder was used as the control group. The extra 1 wt% of resin powders (solid, 40~43 wt% fixed carbon; Wuhan, China) and 4 wt% of phenolic resin (liquid, 45~48 wt% fixed carbon; Shaoxing, China) were used as the binder.

### 2.2. Preparation of the MgO-C Refractories

All the raw materials were evenly mixed, then the mixture was shaped into cylindrical samples (Φ50 mm × H50 mm) under a pressure of 150 MPa after 24 h of staleness. The shaped refractories were subsequently tempered in a heating oven at 110 °C and 200 °C for 24 h. Finally, the low-carbon MgO-C refractories were obtained after firing at 1400 °C for 3 h in a nitrogen atmosphere at a rate of 5 °C/min.

### 2.3. Oxidation Tests

**Figure 1:**
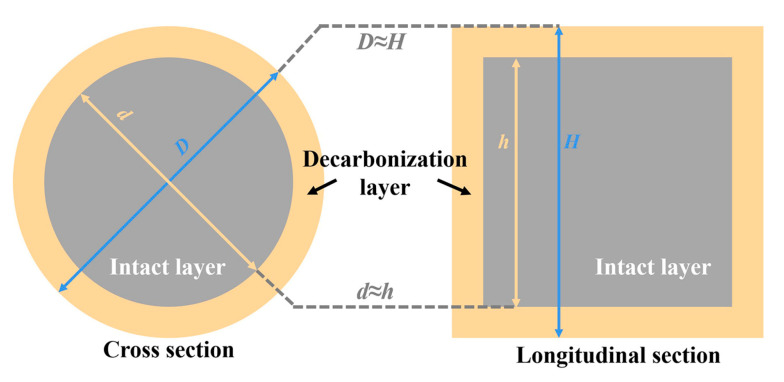
The theoretical profile of the oxidized sample.

The oxidation tests were carried out in an air atmosphere at 1500 °C for 2 h, and the heating rate was 5 °C/min. Figure 1 shows the theoretical profile of the oxidized sample: the outer brown zone was the decarbonization layer, while the central gray zone was considered the intact layer. The physical model for analyzing the oxidation index assumed that the diameter of the sample (*D*) and the intact layer (*d*) were equal to the height of the sample (*H*) and the intact layer (*h*), respectively. The *D* value and *d* value were detected using Image-Pro Plus software (6.0). Then the following oxidation index (O.I.) and rate constant (*k*) were calculated by using Equations (1)–(3) [36].

O.I. = (*D*^2^ − *d*^2^)/*D*^2^ × 100%, (1)

*X* = *V*_o_/*A*_s_ = (*D*^2^*H* − *d*^2^*h*)/(2*D*^2^ + 4*DH*) = (*D*^3^ − *d*^3^)/6*D*^2^, (2)

*k* = *X*^2^/*t*, (3)

where *X*, *V*_o_, *A*_s_, and *t* represent the diffusion depth, volume of the partially oxidized sample, the total open surface area of the partially oxidized sample, and the soaking time of the oxidation test.

### 2.4. Characterizations

Image-Pro Plus software was used to measure the perimeter and area of pores in the decarbonization layer and the thickness of the dense layer, where the thickness value was calculated as the average of 100 measurements. The prepared low-carbon MgO-C refractories (Section 2.2) were crushed to pass through a 0.074 mm sieve, then (about 20 mg) deposited in a corundum crucible and heated from room temperature to 1200 °C (10 °C/min) in flowing air (gas flow 50 mL/min) by the operation of the thermal analyzer (STA 449 C; NETZSCH, Bavaria, Germany). The X-ray diffraction (XRD, X’Pert-Pro-MPD, 40 kV, and 30 mA) with Kα radiation was used to identify the crystalline phases. Before the XRD detection, the sample was ground into fine powders with particle sizes of less than 0.045 mm. The Highscore Plus software (version 3.0) was used for semiquantitative analysis of the generated phases [18]. Scanning electron microscopy (SEM, Nova-400-Nano) combined with an energy-dispersive spectroscopy detector (EDX, Penta FETx3, Oxford) was used to observe the microstructures of samples. The samples for back-scattered SEM observation were vacuum encapsulated in epoxy resin before the testing. Thermodynamic software FactSage (6.2) was employed to calculate the standard Gibbs free energy (Δ*_r_G^θ^*) and the mole contents of the generated gas phases. The equilibrium pressure of the system was 1 atm. A multifunctional high-resolution system (Industrial CT, GE phoenix) was used to detect the distribution, voids, and cracks of the components in the material.

## 3. Results and Discussion

**Figure 2:**
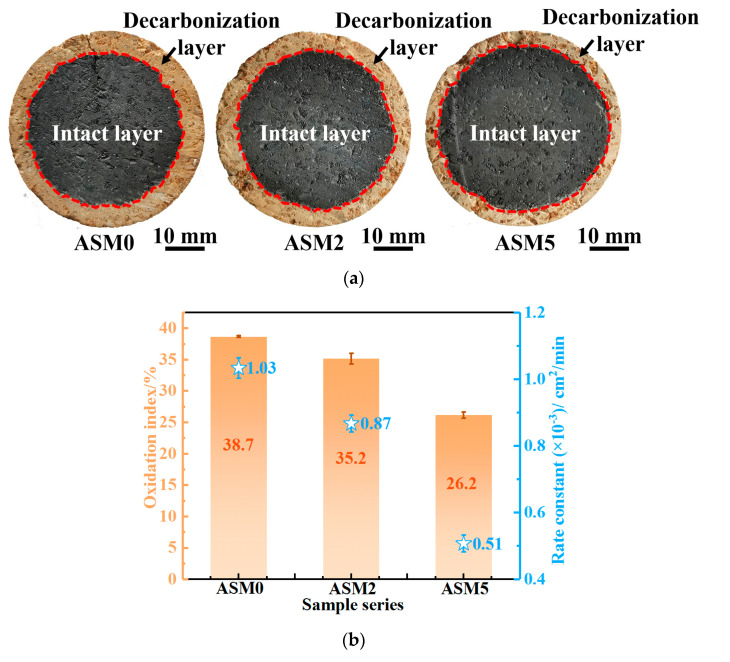
Cross-sectional views (**a**), oxidation index, and rate constant (**b**) of the low-carbon MgO-C refractories fired at 1500 °C.

The cross-sections of the in situ Mg-sialon-enhanced low-carbon MgO-C refractories after the oxidation tests are displayed in Figure 2. The boundary between the decarbonization and intact layer was clear and straightforward (Figure 2a), which indicated that the internal diffusion controlled the oxidation process [9,10,37]. Sample ASM5 showed the highest oxidation resistance, while sample ASM0 showed the lowest oxidation resistance. When compared to sample ASM0, the oxidation index of samples ASM2 and ASM5 decreased from 38.7 ± 0.2% to 35.2 ± 0.9% and 26.2 ± 0.5%, respectively. As shown in Figure 2b, there was a positive relationship between the rate constant and oxidation index. Sample ASM5, with the minimum oxidation index, also had the lowest rate constant, i.e., (0.51 ± 0.03) × 10^−3^ cm^2^/min, thereby indicating an improvement in the oxidation resistance of low-carbon MgO-C refractories with in situ Mg-sialon.

Recent work by Chen et al. [18] has established that the in situ Si_3_N_4_-MgSiN_2_-enhanced MgO-C refractories could be achieved by introducing Fe-containing catalysts. However, the optimized oxidation index of the MgO-Si_3_N_4_-MgSiN_2_-C refractories was as high as 50% after the oxidation tests at 1400 °C for 2 h. In contrast, the MgO-C refractories containing Mg-sialon (ASM5) had a 26.2% oxidation index after the oxidation tests at 1500 °C for 2 h (Figure 2b); these results indicate that, as nitrides, the oxidation resistance of the MgO-C refractories was significantly improved by in situ Mg-sialon.

**Figure 3:**
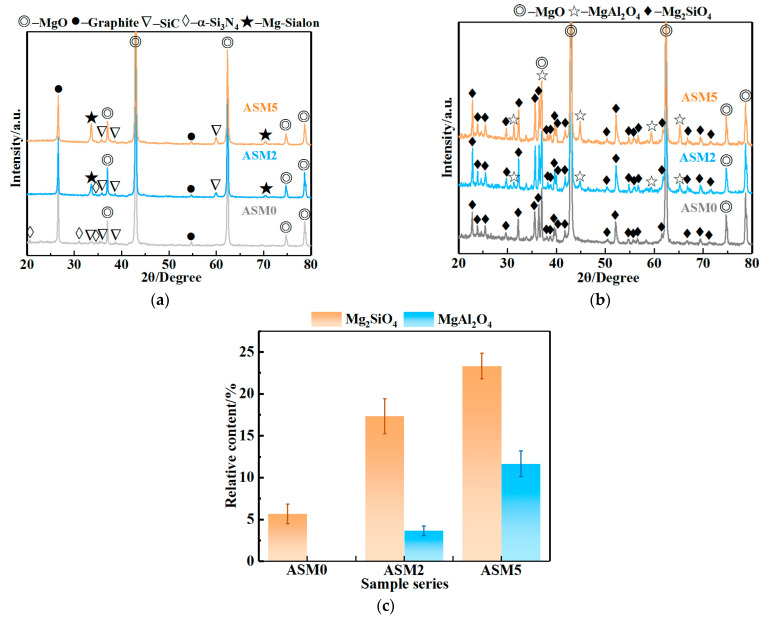
XRD patterns of MgO-C samples before (**a**) and after (**b**) the high-temperature oxidation test and the relative content of Mg_2_SiO_4_ and MgAl_2_O_4_ (**c**) in the oxidation samples.

Figure 3 presents the XRD patterns of the MgO-C samples before and after the high-temperature oxidation test and the relative contents of Mg_2_SiO_4_ and MgAl_2_O_4_. As shown in Figure 3a, the sample without Al_4_SiC_4_ (ASM0) showed graphite, MgO, SiC, and α-Si_3_N_4_ phases. Meanwhile, with the addition of Al_4_SiC_4_, new phases of Mg-Sialon formed in ASM2 and ASM5. It can be determined from the ICSD database that the chemical formula of the as-produced Mg-sialon was Mg_3.29_Si_1.89_Al_2.82_O_4.41_N_4.59_ (PDF # 00-048-1605); meanwhile, the relative intensities of the Mg-sialon and SiC peaks increased with increasing Al_4_SiC_4_ content. After the high-temperature oxidation (Figure 3b), the decarbonization layer contained a newly formed Mg_2_SiO_4_ phase besides MgO in sample ASM0. In addition to graphite oxidation [7], SiC and Si_3_N_4_ were oxidized to produce SiO_2(s)_ [10]. The residual SiO_2(s)_ further reacted with MgO to form Mg_2_SiO_4_ via Equation (4) [18]. Similar to the sialon phase [26], it was believed that MgO, Al_2_O_3_, SiO_2_, C, and N_2_ were formed with the oxidation of Mg-sialon (Equations (5) and (6)). After that, the Al_2_O_3_ would further react with MgO to form MgAl_2_O_4_ (Equation (7)) [7]. It can be seen from Figure 3c that no MgAl_2_O_4_ was found in sample ASM0. In contrast, samples ASM2 and ASM5 showed large amounts of Mg_2_SiO_4_ and MgAl_2_O_4_ after oxidation, and their corresponding oxidation index and rate constant were obviously reduced (Figure 2).

2MgO_(s)_ + SiO_2(s)_ = Mg_2_SiO_4(s)_, (4)

Mg-sialon_(s)_ + O_2(g)_ → MgO_(s)_ + Al_2_O_3(s)_ + SiO_2(s)_ + N_2(g)_ (5)

Mg-sialon_(s)_ + CO_(g)_ → MgO_(s)_ + Al_2_O_3(s)_ + SiO_2(s)_ + N_2(g)_+ C_(s)_ (6)

MgO_(s)_ + Al_2_O_3(s)_ = MgAl_2_O_4(s)_*,* (7)

**Figure 4:**
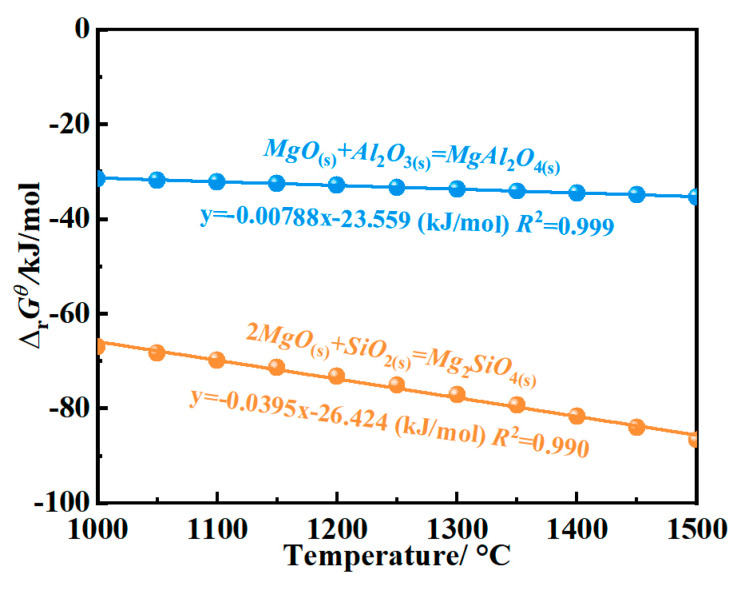
The Δ*_r_G*^θ^ of reactions at 1000~1500 °C.

The Δ*_r_G^θ^* for Equations (5) and (6) could not be calculated due to a lack of thermodynamic data from the Mg-sialon phase (Mg_3.29_Si_1.89_Al_2.82_O_4.41_N_4.59_), and this reaction was given based on the relevant references and the present experimental results [26]. Meanwhile, the thermodynamic calculations for Equations (4) and (7) are shown in Figure 4. The Δ*_r_G^θ^* for these equations was negative at 1000~1500 °C, illustrating that these reactions could proceed toward the right. Based on Equations (5) and (6), Al_2_O_3_ and SiO_2_ would form through the oxidation of Mg-sialon, which favored the occurrence of Equations (4) and (7).

**Figure 5:**
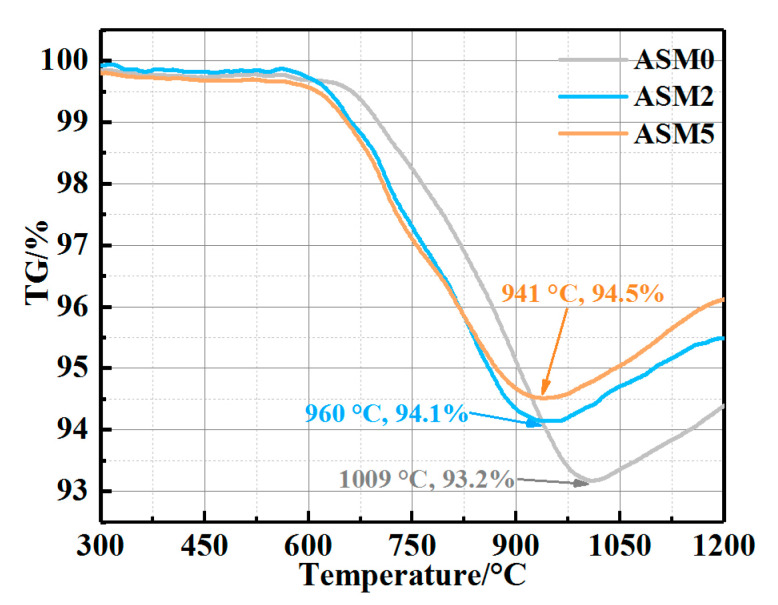
TG curves of the sample matrix in the air.

The change in mass of the samples would be influenced by the oxidation of graphite, carbide, and nitride [10,18,38]. Thermogravimetric (TG) analysis was carried out in order to further investigate the effect of Mg-sialon on the oxidation resistance of the MgO-C refractories. As shown in Figure 5, the TG curves remained stable at 600 °C, and the mass loss observed above 600 °C could be attributed to the oxidation of graphite. As the temperature rose to 941 °C, the TG value of sample ASM5 reached its minimum, i.e., 94.5%. While sample ASM0 had a higher transition temperature (1009 °C) and a lower TG value (93.2%). At 1200 °C, the TG values of sample ASM0, ASM2, and ASM5 were 94.4%, 95.5%, and 96.1%, respectively. This further proved that the content of the generated Mg_2_SiO_4_ and MgAl_2_O_4_ after the oxidation increased with the in situ Mg-sialon.

The onset oxidation temperatures of Si_3_N_4_ and SiC were around 700 °C and 840 °C, respectively [39,40]. Therefore, the oxidation of Si_3_N_4_ was speculated to occur preferentially, followed by the oxidation of SiC. It has been reported that some ion doping in sialon will reduce its initial oxidation temperature [26]. For instance, β-sialon was considered to undergo oxidation at about 900 °C; however, the TG curves of Fe-doped β-sialon showed significant mass gain above 800 °C [26]. Since Mg^2+^ will activate the lattice of sialon, the onset oxidation temperatures of Mg-sialon should also be lower than 900 °C. According to the TG curves (Figure 5), the gain in the mass of the samples with Mg-sialon started at a lower temperature when compared to that of the samples with Si_3_N_4_, and the further oxidation of graphite was restricted due to the preferential oxidation of Mg-sialon.

**Figure 6:**
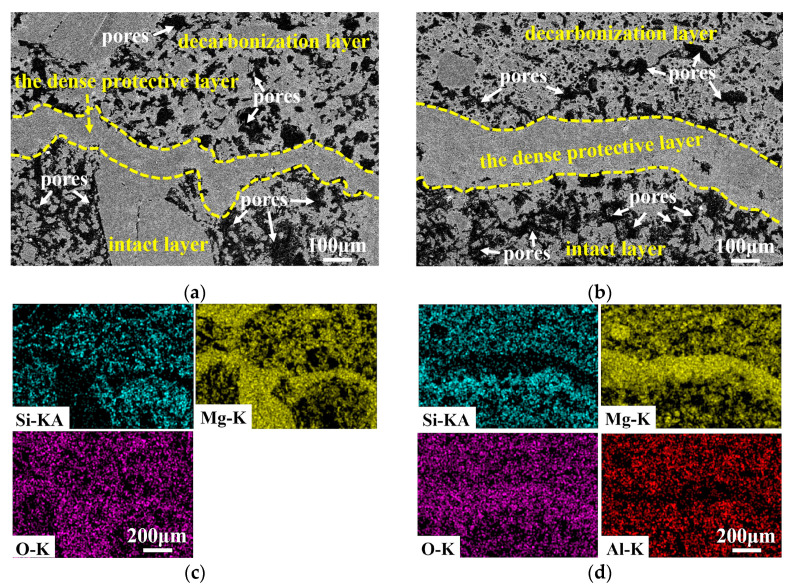
The transition region and the corresponding element distribution of sample ASM0 (**a**,**c**) and ASM5 (**b**,**d**) after the oxidation.

The structure and processes of evolution and transformation have an obvious influence on the oxidation behavior of MgO-C refractories [14]. Figure 6 shows the transition region of samples ASM0 and ASM5 after oxidation. Three zones, including decarbonization layer, the dense protective layer, and the intact layer, could be found in both samples. The dense protective layer formed near the reaction interface and mainly contained Mg and O elements; a trace of Si and Al elements could also be detected, as shown in Figs. 6c and 6d, illustrating that this layer was mainly composed of MgO-Mg_2_SiO_4_ and MgO-Mg_2_SiO_4_-MgAl_2_O_4_ for ASM0 and ASM5, respectively. The dense protective layer mainly resulted from the reaction between the outward diffusion of gaseous Mg vapor and the inward diffusion of oxidizing gas, and Mg vapor originally formed from the indirect oxidation process via Equation (8) at above 1400 °C [41,42]. The combined volume effect of Mg_2_SiO_4_ and MgAl_2_O_4_ could further promote the formation of a protective layer, and the thickness of this layer increased from 86.6 μm to 197.1 μm with the introduction of the Mg-sialon phase. The thicker protective layer efficiently hindered the further inward diffusion of oxygen.

C_(s)_ + MgO_(s)_ = CO_(g)_ + Mg_(g)_, (8)

**Figure 7:**
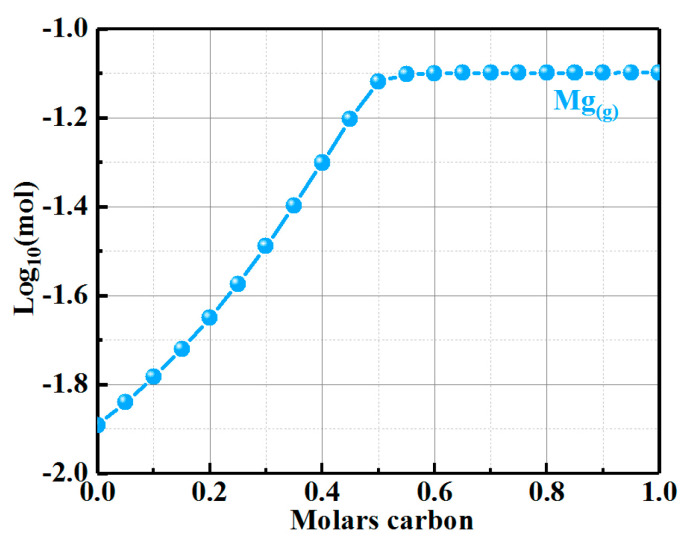
The molar content of Mg gas phase in the MgO-C system at 1500 °C.

The mole content of the Mg vapor in the MgO-C system at 1500 °C was calculated to explore the densification process of the dense protective layer in sample ASM5, as shown in Figure 7. The reactants C_(s)_ and MgO_(s)_ were selected as [A] mole and 1 mole, respectively, where [A] ranges from 0 to 1 mole. As can be seen from the figure, the carbon and Mg vapor had a positive correlation when the carbon content was less than 0.5 mol. Since the mole fraction of the carbon was much lower than 50 % based on the sample composition of the present study, a higher carbon content would accelerate the generation of Mg vapor. As shown in Figure 2 and Figure 5, the preferential oxidation of Mg-sialon resulted in a higher residual carbon content. Therefore, more Mg vapor could form in sample ASM5, which promoted the growth of the dense protective layer.

**Figure 8:**
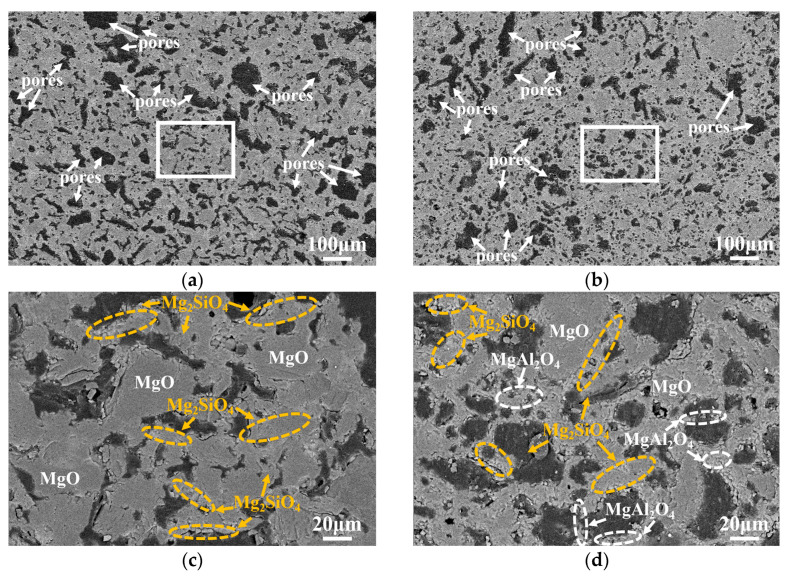

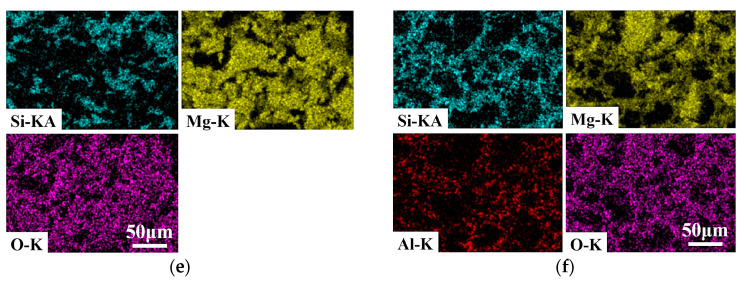
The back-scattered SEM of the oxidation layer and the corresponding element distribution in samples ASM0 (**a**,**c**,**e**) and ASM5 (**b**,**d**,**f**).

The back-scattered SEM and the element distribution of the decarbonization layer in samples ASM0 and ASM5 were further characterized to explore the influence of Mg-sialon on the structure of oxidized MgO-C refractories (Figure 8). The calculated porosity of the decarbonization layer by Image-Pro Plus software for ASM0 (Figure 8a) and ASM5 (Figure 8b) was 21 % and 27 %, and the pore size of the decarbonization layer in ASM5 clearly decreased. The white box areas (Figure 8a,b) were enlarged, as per Figure 8c,d. Compared to ASM0, the MgO particles were closely connected by Mg_2_SiO_4_ and MgAl_2_O_4_ and formed a skeleton structure in ASM5, and this structure could further reduce the oxygen diffusion path.

The oxygen diffusion was significantly dependent on porosity and pore structure [14,15]. In general, the oxidation rate of the sample would be higher when it has an uncomplicated pore structure. The fractal theory was widely used to analyze the pore structure of the refractories [43,44]:

*C*^1/*D*^ = *A*^0.5^, (9)

where *C*, *A*, and *D* represent the perimeter, area, and the fractal dimension of the graphics. In general, 1 ≤ *D* < 2 for plane graphics. If the graphics have poor regularity, i.e., the pore structure of the material is more uncomplicated, the lower *D* value obtains.

**Figure 9:**
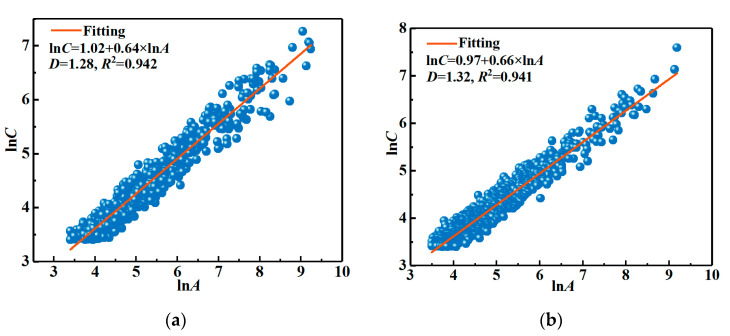
The fractal dimension of the pores and their fitting curve in sample ASM0 (**a**) and ASM5 (**b**).

The Equation (9) can be further derived as:

ln*C* = m + 0.5*D*ln*A.* (10)

where m is a constant. Figure 9 shows the fitting results of the perimeter and area of pores in Figure 8a,b via Equation (10). The determination coefficient (*R*^2^) of the fitting curve was high, indicating that ln*C* and ln*A* had a good linear relationship, and the analytical pores had a fractal structure. Based on the slope of the fitting curve, the fractal dimensions of ASM0 and ASM5 were calculated as 1.28 and 1.32, respectively. This result suggested that the decarbonization layer in sample ASM5 showed a more complex pore structure, resulting in a tortuous path for oxygen diffusion and thus reducing the diffusion rate.

**Figure 10:**
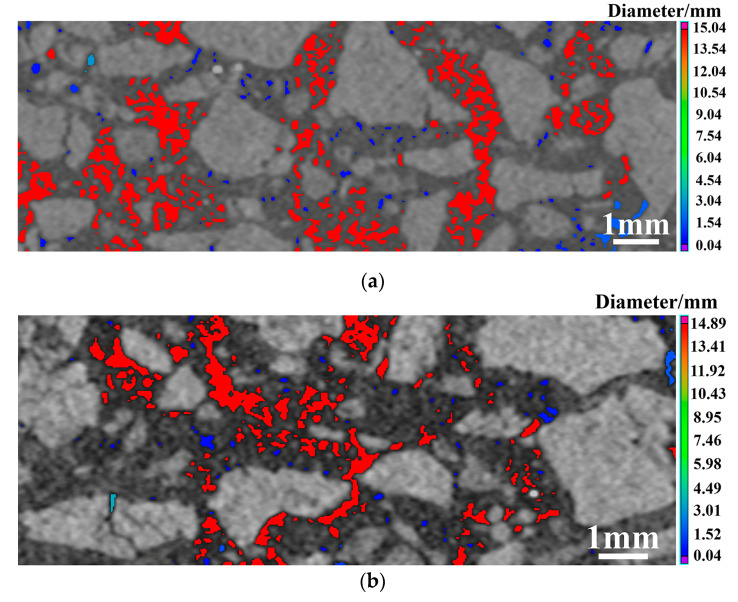
The CT images of the intact layer in the samples without (**a**) and with (**b**) Mg-sialon.

High-resolution industrial CT was successfully applied to scan MgO-C samples to obtain the three-dimensional spatial positions of their constituents [14,18]. Based on the above results, there was a significant change in the pore structure of the transition region and decarbonization layer with the introduction of Mg-sialon, which should also be reflected in the intact layer. A nano CT detection system was used to examine the structure, composition, and defects in the intact layer of the sample with and without Mg-sialon, as shown in Figure 10. The gray parts are the aggregate and matrix, the color parts are the pores, and the different colors indicate various sizes of pore diameter. As shown in the figure, the pores were mainly found between the aggregate and the matrix, and some small, closed pores merged into bigger ones. When compared to Figure 10b, the proportion of red parts in Figure 10a was significantly higher, indicating that there were fewer large pores in the sample with Mg-sialon.

**Figure 11:**
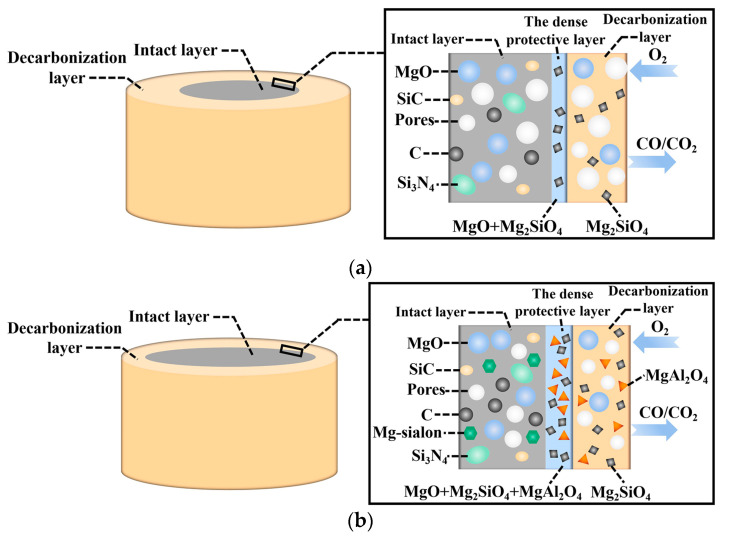
Schematic diagram of the oxidation of the low-carbon MgO-C refractories: (**a**) without Mg-sialon, (**b**) with Mg-sialon.

According to the phase analysis and morphology observations, a schematic diagram for the oxidation of low-carbon MgO-C refractories with in situ Mg-sialon was proposed, as shown in Figure 11. The sample containing Mg-sialon had denser matrix components, which slowed down the oxygen diffusion at the initial stage of oxidation. As the oxidation proceeds, a thicker protective layer was generated with Mg-sialon due to the combined volume effect of Mg_2_SiO_4_ and MgAl_2_O_4_. The reduced porosity and more complex pore structure were also found in the decarbonization layer. Therefore, further oxidation of the in situ Mg-sialon-enhanced low-carbon MgO-C refractories was restricted as the oxygen diffusion path was effectively blocked.

## 4. Conclusions

The low-carbon MgO-C refractories with in situ Mg-sialon were prepared via a nitrification process, adding Al_4_SiC_4_ at 1400 °C for 3 h, and their oxidation behavior was investigated. The sample containing 5 wt% Al_4_SiC_4_ after high-temperature nitridation exhibited the optimal oxidation resistance, with a 26.2% oxidation index at 1500 °C for 2 h. The combined volume effect of Mg_2_SiO_4_ and MgAl_2_O_4_ could promote the formation of a protective layer, and the thickness of this layer increased from 86.6 μm to 197.1 μm with the generation of Mg-sialon. Meanwhile, the sample with Mg-sialon showed a more complex pore structure in the decarbonization layer, leading to a tortuous path for oxygen diffusion and thereby reducing the diffusion rate. Finally, the enhanced oxidation resistance of the low-carbon MgO-C refractories with in situ Mg-sialon could be achieved, as the diffusion of oxygen was effectively delayed by the MgO-Mg_2_SiO_4_-MgAl_2_O_4_ protective layer and the complex diffusion path.

## Figures and Tables

**Table 1:** The composition of the samples (wt%).

Composition	ASM0	ASM2	ASM5
Fused magnesia particles	67	67	67
Fused magnesia powder	26	26	26
Flake graphite	4	4	4
Silicon powder	3	3	3
Resin powder	+1	+1	+1
Phenolic resin	+4	+4	+4
Al_4_SiC_4_ powder	+0	+2	+5

“+” represents the extra addition.

## Data Availability

Data are available on request due to privacy restrictions. The data presented in this study are available on request from the corresponding author.
